# Decoding the Complexity of Hepatocellular Carcinoma: Clinical Challenges and Targeting HuR as a Novel Therapeutic Strategy

**DOI:** 10.3390/livers6040074

**Published:** 2026-08-05

**Authors:** Elizabeth Jones, Natalie Eppler, Forkan Ahamed, Yuxia Zhang

**Affiliations:** Department of Pharmacology, Toxicology and Therapeutics, University of Kansas Medical Center, MS 1018, 3901 Rainbow Boulevard, Kansas City, KS 66160, USA

**Keywords:** hepatocellular carcinoma, clinical challenge, complexity, novel therapeutic strategy, human antigen R (HuR), post-transcriptional regulation

## Abstract

**Background:**

Hepatocellular carcinoma (HCC) is a leading cause of cancer-related mortality worldwide and remains a major therapeutic challenge due to its marked inter- and intratumoral heterogeneity, diverse etiologies, and high propensity for therapeutic resistance. This review summarizes the biological complexity of HCC and current therapeutic challenges, with a particular focus on the RNA-binding protein human antigen R (HuR) as an emerging therapeutic target.

**Methods:**

A comprehensive narrative review of peer-reviewed literature was conducted, focusing on HCC pathogenesis, molecular heterogeneity, tumor microenvironment, mechanisms of therapeutic resistance, and recent advances in treatment. Emphasis was placed on studies investigating the biological functions of HuR and its therapeutic potential in HCC.

**Results:**

HCC progression is driven by complex interactions among genetic, epigenetic, metabolic, and environmental factors, resulting in substantial tumor heterogeneity and variable therapeutic responses. Dysregulated oncogenic signaling and immunosuppressive tumor microenvironment collectively contribute to resistance against current therapies, including multikinase inhibitors and immune checkpoint inhibitors. Although emerging strategies, such as combination immunotherapy, metabolic targeting, epigenetic modulation, and precision medicine, have shown encouraging preclinical and clinical results, their efficacy remains limited by tumor complexity and adaptive resistance. HuR functions as a master post-transcriptional regulator that stabilizes and promotes the translation of numerous mRNAs encoding oncogenic, inflammatory, and pro-survival factors. Accumulating preclinical evidence demonstrates that pharmacological inhibition of HuR suppresses multiple tumor-promoting pathways and enhances therapeutic sensitivity, supporting its potential as a novel therapeutic strategy for HCC.

**Conclusions:**

The biological complexity of HCC necessitates multifaceted, precision-based therapeutic approaches. Although additional HCC-specific mechanistic and translational studies are needed, targeting HuR represents a promising strategy to overcome tumor heterogeneity, therapeutic resistance, and disease progression. Continued integration of molecular profiling, advanced omics technologies, and rational combination therapies will be essential for translating these advances into improved clinical outcomes for patients with HCC.

## 1. Introduction

Liver cancer is the third leading cause of cancer-related deaths worldwide and the sixth most common malignancy, with hepatocellular carcinoma (HCC) accounting for 85–90% of primary liver cancer cases [1,2]. HCC arises from hepatocytes, the main functional cells of the liver, with the remaining 10–15% of primary liver cancers originating from cholangiocytes as cholangiocarcinoma [3–7]. If detected early, surgical resection, chemotherapy, and radiation are effective treatments for HCC [5]. However, a significant challenge in HCC treatment is the silent nature of the disease during its early stages. HCC often presents with vague symptoms or is asymptomatic until it progresses to an advanced stage [8]. Patients with late-stage HCC have a higher level of recurrence, poor prognosis, and an increased chance of metastasis, even with traditional treatments [8]. Furthermore, only approximately 20–30% of patients with HCC are eligible for potentially curative treatments at diagnosis, leaving the majority unsuitable for surgical resection or liver transplantation due to advanced tumor burden, impaired hepatic functional reserve, or both [9,10].

The global burden of HCC demonstrates marked geographic heterogeneity. The highest age-standardized incidence rates are observed in Eastern Asia (14.8–17.8 per 100,000 population), Northern Africa (13.2–15.2 per 100,000), and Southeast Asia (9.5–13.7 per 100,000) [7,11]. By comparison, Europe and North America have intermediate incidence rates (approximately 5–8 per 100,000), whereas Latin America and Oceania generally experience lower incidence, although substantial regional variability exists within these regions [7,11]. Historically, chronic hepatitis B virus (HBV) and hepatitis C virus (HCV) infections have been the predominant drivers of HCC and remain major etiological factors in many regions of Asia and Africa. Widespread HBV vaccination programs and the advent of effective antiviral therapies have substantially reduced the burden of viral hepatitis, leading to a shift in the global etiology of HCC [12]. This transition has been accompanied by the rising prevalence of obesity, type 2 diabetes, and metabolic syndrome, resulting in a marked increase in metabolic dysfunction-associated steatotic liver disease (MASLD), formerly known as non-alcoholic fatty liver disease (NAFLD), as a leading cause of HCC [12]. Consequently, the global burden of liver cancer is expected to continue increasing, with the annual incidence projected to rise by 55% and more than 1.3 million liver cancer-related deaths anticipated by 2040 [13]. Given the substantial regional variation in disease etiology, the high frequency of late-stage diagnosis, and the limited availability of curative treatment options, HCC remains a complex and formidable clinical challenge. Addressing this challenge will require a deeper understanding of the molecular mechanisms underlying hepatocarcinogenesis to facilitate the identification of novel therapeutic targets and reliable, non-invasive biomarkers for early diagnosis and personalized treatment.

## 2. HCC Staging Classification

The classification of HCC has evolved considerably to better reflect regional differences in disease etiology, advances in tumor biology, and the growing need for personalized treatment strategies. Modern HCC staging systems integrate tumor burden, liver function, and patient performance status to predict prognosis and guide therapeutic decision-making. Unlike most solid tumors, HCC staging must account for both tumor progression and the underlying liver disease, making accurate staging essential for selecting appropriate treatment and estimating clinical outcomes.

One of the earliest staging systems, the Okuda classification, incorporates tumor size, the presence of ascites, and serum albumin levels to assess disease severity [1]. Subsequently, several region-specific staging systems were developed, including the Japan Integrated Staging (JIS) score, Hong Kong Liver Cancer (HKLC) classification, and Cancer of the Liver Italian Program (CLIP) score, each integrating different combinations of tumor characteristics, liver function, and prognostic biomarkers. The JIS score combines tumor stage with liver function to improve prognostic prediction [1], whereas the HKLC system integrates tumor stage, liver function, and serum biomarkers to facilitate treatment selection [14,15]. The CLIP score incorporates tumor morphology, serum alpha-fetoprotein (AFP) levels, and liver function as defined by the Child-Pugh classification to estimate patient prognosis [1,16].

The Tumor-Node-Metastasis (TNM) staging system, developed by the American Joint Committee on Cancer (AJCC) and the Union for International Cancer Control (UICC), focuses primarily on tumor size, vascular invasion, lymph node involvement, and distant metastasis, making it particularly valuable for pathological staging following surgical resection [1,17]. In contrast, the Barcelona Clinic Liver Cancer (BCLC) staging system (Table 1) has become the most widely adopted framework in clinical practice; combining tumor stage, liver function (Child-Pugh class), and patient performance status according to the Eastern Cooperative Oncology Group (ECOG) to provide both prognostic assessment and treatment recommendations [1,18–21]. The BCLC system categorizes HCC into five stages (0, A, B, C, and D), guiding management from curative therapies for very early- and early-stage disease to locoregional treatment, systemic therapy, and palliative care for advanced disease. Recent updates to the BCLC algorithm have further incorporated additional prognostic indicators, including serum AFP, the albumin-bilirubin (ALBI) grade, and the Model for End-Stage Liver Disease (MELD) score, to refine patient stratification and facilitate individualized treatment planning [18,22].

Although these staging systems differ in their specific criteria, they all evaluate prognosis using three principal components: tumor burden, liver function, and patient performance status [16]. Tumor burden—including tumor size, number of lesions, vascular invasion, and distant metastasis—is a major determinant of prognosis, with larger and multifocal tumors associated with significantly poorer survival [8,23]. Vascular invasion, particularly portal vein invasion, is a strong predictor of recurrence, metastatic spread, and reduced overall survival [1,8]. Liver function remains equally important because it directly influences both prognosis and treatment eligibility. The Child-Pugh score, which incorporates serum bilirubin, albumin, prothrombin time, ascites, and hepatic encephalopathy, is widely used to assess hepatic reserve, with higher scores indicating worse liver function and poorer clinical outcomes [8,16,24]. Additional indicators, including portal hypertension, the ALBI grade, and the MELD score, further improve assessment of hepatic functional reserve and patient prognosis [8,25,26]. Finally, the ECOG performance status provides an objective measure of functional capacity and treatment tolerance, with declining performance status strongly associated with reduced survival [8,16].

Despite substantial improvements in HCC staging, distinguishing early-stage from advanced disease remains one of the greatest clinical challenges with prognosis and treatment options differing dramatically between these groups. Patients diagnosed with very early- or early-stage HCC may achieve long-term survival or cure through surgical resection, liver transplantation, or local ablative therapies. In contrast, most patients are diagnosed at intermediate or advanced stages, when curative treatment is no longer feasible and systemic therapies provide only modest survival benefits, contributing to an overall 5-year survival rate of approximately 18% [5]. These limitations underscore the urgent need for earlier diagnosis, improved risk stratification, and a deeper understanding of the molecular mechanisms driving hepatocarcinogenesis to facilitate the development of reliable biomarkers and more effective targeted therapies.

## 3. HCC: A Multifactorial and Complex Disease

**Figure 1.**
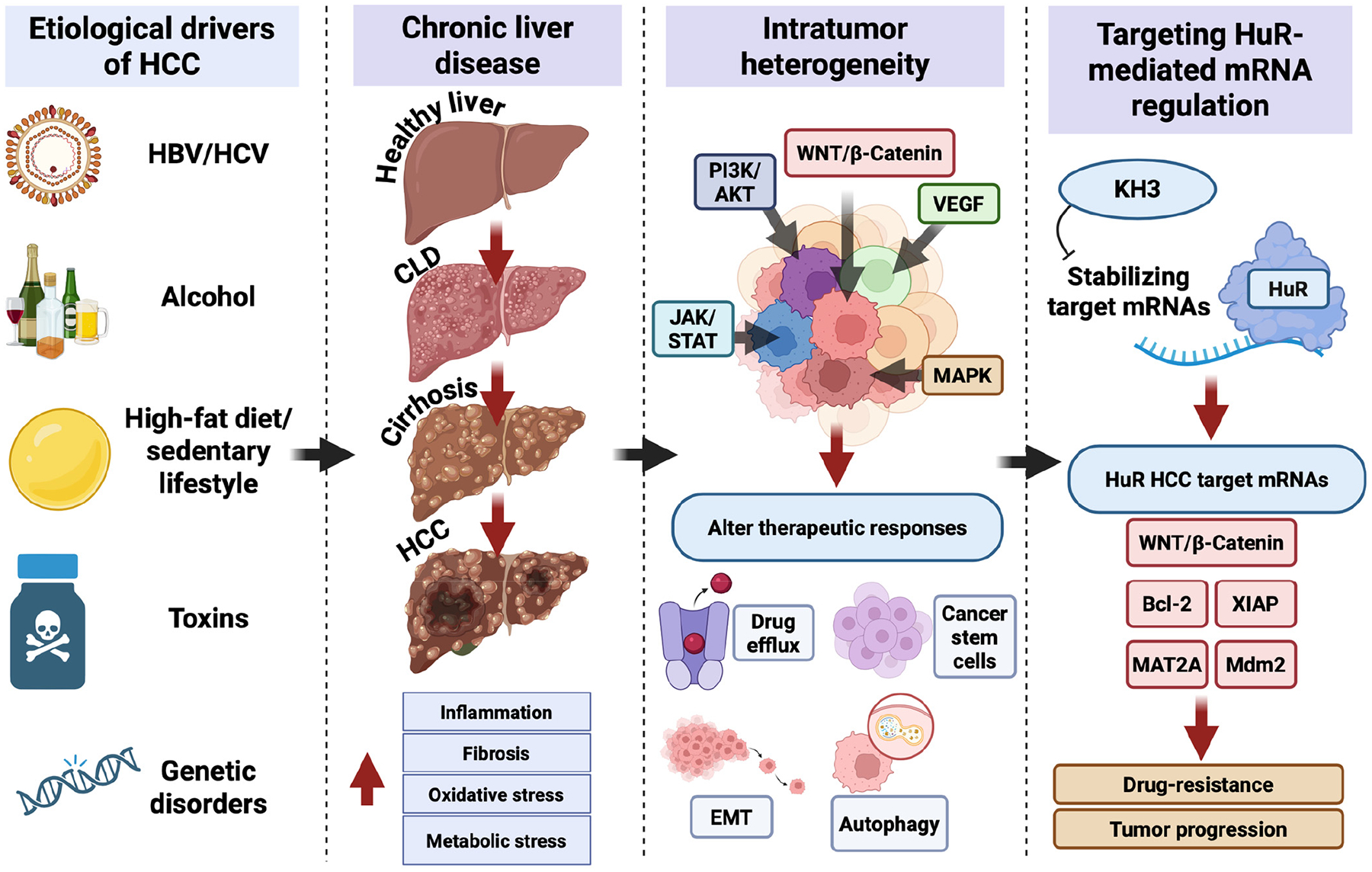
Schematic overview of etiological risk factors, intratumoral heterogeneity, and therapeutic targeting of HuR in hepatocellular carcinoma (HCC). HCC arises from diverse and often coexisting risk factors, including chronic viral infections (hepatitis B and C), alcohol-associated liver disease (ALD), metabolic dysfunction–associated steatotic liver disease (MASLD), aflatoxin exposure, and genetic susceptibility. Collectively, these factors synergize to drive chronic inflammation, fibrosis, oxidative stress, and metabolic dysfunction, establishing a permissive hepatic microenvironment that promotes disease progression from chronic liver disease (CLD) to cirrhosis and ultimately supports HCC initiation and progression. Genomic and epigenomic studies reveal extensive heterogeneity in HCC, characterized by alterations in multiple signaling pathways, including Janus Kinase (JAK)/signal transducer and activator of transcription (STAT), phosphatidylinositol 3-kinase (PI3K)/protein kinase B (AKT), Wingless/Integrated (Wnt)/β-catenin, vascular endothelial growth factor (VEGF), and mitogen-activated protein kinase (MAPK), which contribute to variable therapeutic responses. Additional layers of complexity arise from cancer stem cells (CSCs), dysregulated drug efflux, epithelial–mesenchymal transition (EMT), and autophagy, all of which promote resistance and disease progression. Hu antigen R (HuR), encoded by the ELAV-like RNA-binding protein 1 (*ELAVL1*) gene, regulates a broad network of mRNAs that drive hepatocarcinogenesis. Pharmacologic inhibition of HuR, such as with KH-3, disrupts HuR–mRNA interactions, leading to destabilization of oncogenic transcripts, including components of Wnt/β-catenin signaling, B-cell lymphoma 2 (*BCL-2*), X-linked inhibitor of apoptosis protein (*XIAP*), Methionine adenosyltransferase 2A (*MAT2A*), and Murine double minute 2 (*MDM2*), thereby suppressing drug resistance and tumor progression.

HCC arises from a wide range of risk factors, including chronic viral infections (Hepatitis B and C), alcohol-associated liver disease (ALD), MASLD, aflatoxin exposure, and genetic susceptibility (Figure 1). These factors often coexist and interact synergistically, driving chronic inflammation, fibrosis, and cirrhosis, creating a permissive environment for tumor development. Convergence of diverse etiologies results in substantial genetic, epigenetic, and phenotypic heterogeneity, resulting in a multifactorial and highly complex disease. This complexity contributes to variability in disease progression, therapeutic responsiveness, and clinical outcomes, posing significant challenges for effective management and treatment.

### 3.1. Hepatitis B and C

Chronic HBV and HCV infections have historically been the predominant causes of HCC worldwide, although their relative contributions vary geographically. HBV remains the leading etiologic factor in Asia and Africa, accounting for approximately 60% of HCC cases, whereas it contributes to only about 20% of cases in Western countries [8,27]. In contrast, HCV-related HCC is more prevalent in Europe and North America [8]. Globally, HBV and HCV account for approximately 54% and 31% of HCC cases, respectively, affecting an estimated 400 million and 170 million individuals worldwide [8,28,29].

Both HBV and HCV promote hepatocarcinogenesis through persistent hepatocyte injury, chronic inflammation, oxidative stress, and compensatory liver regeneration, creating a pro-fibrotic and tumorigenic microenvironment [30]. However, the molecular mechanisms underlying HBV- and HCV-induced HCC differ substantially. HBV integrates its DNA into the host genome, leading to genomic instability, dysregulated gene expression, and uncontrolled hepatocyte proliferation. In addition, the HBV-encoded X protein (HBx) promotes hepatocarcinogenesis by inducing reactive oxygen species (ROS) production, mitochondrial dysfunction, inflammatory signaling, and activation of multiple oncogenic pathways [31–33]. Interestingly, HBx expression is frequently retained in HBV-related HCC tissues, often in the form of C-terminally truncated variants with enhanced oncogenic activity [34,35]. Moreover, HBV-associated HCC can arise in the absence of advanced fibrosis or cirrhosis, supporting a direct oncogenic role for viral DNA integration and HBx-mediated dysregulation of host signaling pathways [36,37].

Unlike HBV, HCV does not integrate into the host genome but promotes hepatocarcinogenesis through chronic inflammation, epigenetic alterations, and oxidative stress. HCV infection induces aberrant DNA methylation and other epigenetic modifications that alter gene expression and contribute to malignant transformation [38,39]. Furthermore, the HCV core protein disrupts the mitochondrial electron transport chain and depletes intracellular and mitochondrial glutathione (GSH), resulting in excessive ROS production and mitochondrial dysfunction [40,41]. Consistent with these observations, transgenic mice expressing either the HCV core protein or the complete HCV genome develop HCC through mechanisms involving oxidative stress and iron overload, even in the absence of significant inflammation [42–45]. The risk of HCC also varies according to viral genotype. HBV genotype C is associated with a higher risk of HCC than genotype B, whereas HCV genotype 1b has been associated with greater HCC risk than non-1b genotypes and HCV genotype 3 with greater risk than genotype 1 [46–49].

Overall, HBV and HCV promote HCC through both shared and distinct mechanisms. Although both viruses induce oxidative stress and chronic liver injury, HBV primarily drives hepatocarcinogenesis through viral DNA integration, genomic instability, and the oncogenic activity of HBx, whereas HCV predominantly promotes tumor development through chronic inflammation, epigenetic reprogramming, and mitochondrial dysfunction. Importantly, viral proteins from both HBV and HCV possess direct oncogenic properties that can promote HCC independently of advanced fibrosis or cirrhosis. Nevertheless, the global burden of virus-associated HCC has declined substantially owing to widespread HBV vaccination and the remarkable success of direct-acting antiviral therapies for HCV, shifting the epidemiology of HCC toward MASLD and ALD in many regions of the world [16,50,51].

### 3.2. Lifestyle Factors

Lifestyle-related exposures are a major risk factor for HCC development. Excessive alcohol consumption is a well-established risk factor for HCC, which often manifests as ALD [8,16,27,52]. ALD spans a clinical spectrum from steatosis to cirrhosis and is a significant risk factor for HCC, especially in combination with other factors, such as viral hepatitis [53,54]. ALD accounts for approximately 20–25% of HCC cases, with approximately 1–3% of alcoholic cirrhosis cases developing into HCC every year [55–58]. Alcohol cessation remains the cornerstone of management and is associated with an estimated 6–7% annual reduction in cancer risk, although adherence varies [55]. Mechanistically, ethanol induces hepatocyte toxicity through oxidative stress, inflammation, apoptosis, and DNA damage with impaired repair capacity [53,59,60]. Acetaldehyde is a carcinogenic ethanol metabolite that forms DNA and protein adducts and acts synergistically with ethanol-inducing ROS to exacerbate oxidative injury [61]. As with a variety of cancers, cigarette smoking may act as a cofactor in HCC development, potentially increasing ROS levels and acting synergistically with other risk factors to promote hepatocarcinogenesis [62,63].

Lifestyle factors, such as poor diet and a lack of physical activity, also contribute to the development of metabolic disorders and are a growing concern globally, particularly in western countries [8,16,54]. In the last few decades, there has been an observable rise in obesity and diabetes leading to an increase in MASLD, which can progress to metabolic dysfunction-associated steatohepatitis (MASH) and cirrhosis [54]. The global prevalence of MASLD is about 25–30%, with MASH having a global prevalence of approximately 5.27% [64–67]. Patients with MASH and cirrhosis have an 80 times greater risk for HCC development than those with non-cirrhotic MASLD [68]. MASLD progression to MASH also increases the annual HCC incidence rates by 12-fold [12,64]. As chronic liver disease (CLD) progresses, excessive hepatic steatosis, inflammation, fibrosis, and ultimately scar tissue formation contribute to HCC development. Furthermore, metabolic syndromes, such as obesity, insulin resistance, dyslipidemia, and diabetes mellitus, have also been linked to an increased risk of HCC, owing to insulin resistance and chronic inflammation [64,69–71]. Overall, as CLD advances, the risk of HCC increases in parallel, underscoring the critical importance of modifying lifestyle factors in order to reduce the burden of disease.

### 3.3. Environmental Exposures and Genetic Disorders

In addition to viral hepatitis and metabolic liver disease, environmental exposures and inherited disorders represent important risk factors for HCC [8,72]. Among environmental carcinogens, aflatoxin is one of the best-established hepatotoxic agents. Produced primarily by *Aspergillus flavus* and *Aspergillus parasiticus*, aflatoxins commonly contaminate maize, other cereal grains, peanuts, and tree nuts, particularly in warm and humid regions and under inadequate preharvest or postharvest storage conditions [73,74]. Following hepatic metabolism by cytochrome P450 enzymes (primarily CYP1A2 and CYP3A4), aflatoxin is converted to the highly reactive metabolite aflatoxin B1–8,9-epoxide, which forms DNA adducts, induces mutagenesis, and promotes hepatocarcinogenesis [75].

Several inherited metabolic disorders also predispose individuals to HCC by promoting chronic liver injury, fibrosis, and cirrhosis [8]. Hereditary hemochromatosis, characterized by excessive iron accumulation, promotes oxidative stress, lipid peroxidation, and DNA damage, thereby facilitating malignant transformation [76]. Wilson’s disease, caused by impaired copper metabolism, results in hepatic copper accumulation that induces oxidative stress, mitochondrial dysfunction, and activation of six transmembrane epithelial antigens of the prostate 2 (STEAP2) and downstream mitogen-activated protein kinase (MAPK) signaling pathways, including c-Jun N-terminal kinase (JNK) and p38, thereby promoting inflammation and hepatocarcinogenesis [77]. Likewise, alpha-1 antitrypsin deficiency leads to the accumulation of misfolded alpha-1 antitrypsin protein within hepatocytes, causing chronic hepatocellular injury, fibrosis, and an increased risk of HCC [78].

Autoimmune liver diseases, including autoimmune hepatitis (AIH), primary biliary cholangitis (PBC), and primary sclerosing cholangitis (PSC), also increase the risk of HCC through persistent immune-mediated liver injury, chronic inflammation, and progressive fibrosis [58,79,80]. A multicenter retrospective cohort study from Europe and Canada reported that cirrhosis was present in approximately 20.5% of patients with AIH and was associated with a threefold increased risk of HCC [80]. Furthermore, patients with concurrent AIH and PSC exhibited an approximately fivefold higher risk of developing HCC [80].

Collectively, these diverse etiologic factors converge on common pathogenic mechanisms that promote hepatocarcinogenesis. Persistent hepatocyte injury, chronic inflammation, oxidative stress, genomic instability, dysregulated cellular signaling, and progressive fibrosis drive the transition from chronic liver disease to cirrhosis and ultimately HCC. This convergence of multiple pathogenic pathways highlights the biological complexity of HCC and contributes to the limited efficacy of current therapies, particularly in patients with advanced or treatment-resistant disease.

## 4. Current Therapeutics

Despite substantial advances in surveillance, diagnosis, and treatment, the overall 5-year survival rate for patients with HCC remains at approximately 18%, increasing to 31% among those diagnosed at an early stage [13]. This poor prognosis is largely attributable to the frequent diagnosis of HCC at intermediate or advanced stages, when potentially curative interventions—including surgical resection, liver transplantation, local ablative therapies, and radiation therapy—are no longer feasible or offer limited clinical benefit. Clinical management is further complicated by the remarkable molecular and cellular complexity of HCC, which arises from its diverse etiologies and is characterized by extensive inter- and intratumoral heterogeneity. Consequently, tumors from different patients—and even multiple lesions within the same patient—can exhibit distinct genetic, epigenetic, metabolic, and immunological features that influence disease progression and therapeutic response [6,81–83]. This biological heterogeneity contributes to therapeutic resistance, tumor recurrence, metastasis, and poor clinical outcomes, representing one of the greatest barriers to effective HCC treatment.

For patients who are not eligible for curative therapies, systemic treatment has become the standard of care. Current first-line systemic treatment options for unresectable or advanced HCC include the multikinase inhibitors sorafenib or lenvatinib and immune checkpoint inhibitor-based combinations such as atezolizumab plus bevacizumab, tremelimumab plus durvalumab, and nivolumab plus ipilimumab [5,84–87]. Although these therapies have significantly improved survival compared with earlier standards of care, their overall efficacy remains limited with only a small subset of patients achieving an objective response, while many exhibit primary resistance or eventually develop acquired resistance to multikinase inhibitors and immunotherapies [88–90]. Furthermore, treatment efficacy is influenced by multiple clinical and molecular factors, including disease stage, hepatic functional reserve, performance status, tumor burden, viral etiology, serum AFP concentration, bleeding risk, contraindications to immunotherapy, and expected treatment-related toxicities.

To provide readers with a concise overview of currently available systemic treatment options, Table 2 summarizes therapies with U.S. Food and Drug Administration (FDA)-approved HCC-specific indications as of July 2026. To avoid discrepancies among international regulatory agencies, investigational agents and tumor-agnostic therapies without HCC-specific FDA approval are not included. Despite these therapeutic advances, durable clinical responses remain limited, underscoring the need for novel treatment strategies that target the molecular drivers of HCC and overcome the biological complexity underlying therapeutic resistance.

### 4.1. Transarterial Chemoembolization (TACE)

TACE is the standard locoregional therapy for patients with intermediate-stage HCC who are not candidates for surgical resection, liver transplantation, or local ablative therapies. The procedure involves selective catheterization of the hepatic artery to deliver high concentrations of chemotherapeutic agents directly to the tumor, followed by arterial embolization to induce ischemia and prolong local drug retention [5,91]. By combining targeted chemotherapy with vascular occlusion, TACE promotes tumor necrosis while minimizing systemic drug exposure [92,93]. In certain cases, TACE may also be combined with local ablative therapies to improve tumor control when curative treatment is not feasible [94].

Conventional TACE employs a lipiodol-based emulsion as the drug carrier; however, inconsistent lipiodol deposition and heterogeneous intratumoral drug distribution often result in variable therapeutic efficacy. To overcome these limitations, drug-eluting bead TACE (DEB-TACE) was developed to provide sustained and controlled intratumoral drug release while reducing systemic drug exposure. Nevertheless, drug release from DEBs remains influenced by microsphere size, composition, and tumor vascularity, resulting in incomplete drug release and variable therapeutic responses [95–97].

Despite its established clinical benefit, TACE is associated with several important limitations. Post-embolization syndrome, characterized by abdominal pain, fever, nausea, vomiting, and transient elevations in liver enzymes, occurs in approximately 47.7% of treated patients [98]. DEB-TACE has also been associated with an increased risk of hepatic injury and biliary complications [99]. Moreover, ischemia induced by embolization creates a hypoxic tumor microenvironment that stimulates vascular endothelial growth factor (VEGF) expression and angiogenic signaling, promoting tumor revascularization, recurrence, and disease progression [100]. Consequently, although TACE effectively controls localized disease, its long-term efficacy as a standalone treatment remains limited by tumor recurrence and the development of therapeutic resistance.

To overcome these limitations, TACE is increasingly combined with systemic therapies, including molecularly targeted agents and immune checkpoint inhibitors, with the goal of improving local tumor control and prolonging survival. However, randomized phase II and III clinical trials have yielded mixed results, with some studies demonstrating no significant survival benefit, whereas others reported an improvement in overall survival of approximately six months [91,100,101]. More recently, combination therapy with TACE, lenvatinib, and programmed cell death protein 1 (PD-1) inhibitors has shown encouraging clinical activity in patients with intermediate- to advanced-stage HCC by reducing serum AFP levels, enhancing antitumor immune responses, and modulating pro-tumorigenic cytokine signaling without substantially increasing treatment-related toxicity [102]. Nevertheless, concerns regarding acquired resistance to chemotherapies and with the marked biological heterogeneity of HCC, continue to limit the widespread implementation of these combination strategies.

### 4.2. Multikinase Inhibitors (MKIs)

HCC is a highly vascularized malignancy that relies heavily on angiogenic signaling, particularly the VEGF/VEGF receptor (VEGFR) axis, to sustain tumor growth, invasion, and metastasis [103]. Consequently, multikinase inhibitors (MKIs), which simultaneously target angiogenesis and multiple oncogenic signaling pathways, have become a cornerstone of first-line systemic therapy for advanced HCC [9,14,87,104].

Sorafenib was the first systemic therapy approved for advanced HCC and marked a major milestone in the management of unresectable disease. Sorafenib inhibits multiple receptor tyrosine kinases involved in tumor angiogenesis, including VEGFR1–3, platelet-derived growth factor receptor (PDGFR), and KIT proto-oncogene receptor tyrosine kinase (KIT), as well as the serine/threonine kinases RAF-1 and BRAF, thereby suppressing tumor cell proliferation, angiogenesis, and survival signaling [105,106]. Although sorafenib significantly improved the treatment landscape for advanced HCC, its clinical benefit remains modest, extending overall survival by only approximately 2.3–2.8 months [14,107–109]. Moreover, treatment is frequently associated with adverse events, including anorexia, hepatotoxicity, hypertension, bleeding complications, dermatologic toxicities, and arterial thromboembolic events, which often limit long-term use [110–114].

The limited efficacy of sorafenib is largely attributable to both intrinsic and acquired resistance. Multiple mechanisms contribute to resistance, including activation of compensatory oncogenic signaling pathways, enhanced angiogenesis, increased drug efflux, metabolic reprogramming, and adaptive changes within the tumor microenvironment [115,116]. In sorafenib-treated patients with HCC, oncogenic alterations in phosphoinositide 3-kinase (PI3K)-AKT-mechanistic target of rapamycin (mTOR) signaling pathway were associated with significantly poorer clinical outcomes, including a lower disease control rate (8.3% vs. 40.2%), shorter median progression-free survival (1.9 vs. 5.3 months), and reduced median overall survival (10.4 vs. 17.9 months) [117]. In addition, substantial interpatient variability in pharmacokinetics—sometimes referred to as “pharmacokinetic resistance”—may further contribute to inconsistent therapeutic responses, with up to 50% variability observed among individual patient responses [117–120].

Lenvatinib, another orally administered multitarget receptor tyrosine kinase inhibitor, is approved as a first-line treatment for unresectable HCC and has demonstrated non-inferiority to sorafenib while providing improved progression-free survival [121,122]. Lenvatinib inhibits VEGFR1–3, fibroblast growth factor receptors (FGFR1–4), PDGFRα, KIT, and rearranged during transfection (RET), thereby suppressing angiogenesis and tumor proliferation [122,123]. Through inhibition of these receptors, lenvatinib also attenuates several downstream oncogenic pathways, including RAS-RAF-MEK-ERK and PI3K-AKT pathways, thereby limiting tumor-cell proliferation, survival, invasion, and metabolic adaptation [124,125]. Lenvatinib can also modulate JAK–STAT signaling in a context-dependent manner, particularly through effects on FGFR signaling and the tumor immune microenvironment [125]. Common treatment-related adverse effects include hypertension, proteinuria, fatigue, and arterial thromboembolic events [121,122].

Despite its favorable clinical activity, resistance to lenvatinib also develops rapidly, limiting durable responses. Mechanistically, lenvatinib resistance has been linked to activation of the epidermal growth factor receptor (EGFR)-STAT3-ATP-binding cassette subfamily B member 1 (ABCB1) signaling axis, which enhances drug efflux and reduces intracellular drug accumulation [126]. Preclinical studies have demonstrated that combining lenvatinib with EGFR inhibitors, such as erlotinib, can suppress STAT3-ABCB1 signaling and partially restore drug sensitivity [126]. Although this strategy has shown promise in experimental HCC models and EGFR inhibition is clinically established in other malignancies, including non-small-cell lung cancer [127], its therapeutic efficacy in HCC remains to be validated in prospective clinical trials.

Collectively, MKIs have significantly improved the management of advanced HCC by targeting multiple angiogenic and proliferative signaling pathways. However, the development of intrinsic and acquired resistance, treatment-related toxicities, and substantial interpatient variability continue to limit their long-term efficacy. These challenges underscore the need for rational combination therapies and novel therapeutic approaches that target complementary molecular mechanisms underlying HCC progression and therapeutic resistance.

### 4.3. Immune Checkpoint Inhibitors

The limited efficacy of MKIs and the emergence of therapeutic resistance has driven the development of immunotherapy for advanced HCC. Immune checkpoint inhibitors restore antitumor immunity by disrupting inhibitory signaling pathways that suppress T-cell activation within the tumor microenvironment. These antibodies targeting the PD-1/programmed cell death ligand 1 (PD-L1) axis have become a cornerstone of systemic HCC therapy [128,129]. PD-1 is an inhibitory receptor expressed on activated T cells, whereas PD-L1 is frequently upregulated on tumor cells and immune cells within the HCC microenvironment. Engagement of PD-1 by PD-L1 suppresses T-cell activation, proliferation, and cytokine production, enabling tumor immune evasion [130–134]. Monoclonal antibodies targeting PD-1, including nivolumab, disrupt this interaction and restore antitumor T-cell activity. Nivolumab monotherapy has demonstrated an objective response rate of approximately 18.2% in patients with advanced HCC [135,136], although the duration and magnitude of clinical benefit vary considerably among responders.

Despite these advances, only a subset of patients achieves durable responses to PD-1 blockade [136]. Emerging evidence indicates that the molecular heterogeneity of HCC contributes substantially to immunotherapy resistance. For example, activation of the Wingless/Integrated (Wnt)/β-catenin signaling pathway has been associated with T-cell exclusion and primary resistance to anti-PD-1 therapy [137]. Likewise, patients with MASH-associated HCC appear to derive less clinical benefit from immune checkpoint blockade and, in some experimental settings, may even exhibit paradoxical tumor progression following anti-PD-1 therapy [12]. Although immune checkpoint inhibitors are generally better tolerated than conventional chemotherapy, they can induce immune-related adverse events (irAEs), including pneumonitis, colitis, endocrinopathies such as autoimmune diabetes, hepatitis, and dermatologic toxicities [138–141]. Early recognition and prompt management are essential to minimize treatment-related morbidity and mortality.

Given the heterogeneous responses to immune checkpoint blockade, combination immunotherapy has become a major focus of HCC treatment. The combination of the PD-L1 inhibitor atezolizumab and the VEGF inhibitor bevacizumab has demonstrated superior overall and progression-free survival compared with sorafenib and is now a preferred first-line treatment for patients with unresectable HCC [86]. Mechanistically, atezolizumab restores T-cell-mediated antitumor immunity by blocking PD-L1, whereas bevacizumab inhibits VEGF-driven angiogenesis and helps normalize the tumor vasculature, thereby enhancing immune cell infiltration into the tumor microenvironment [86,87]. Other immune checkpoint pathways have also emerged as promising therapeutic targets. Cytotoxic T-lymphocyte-associated protein 4 (CTLA-4) inhibitors, including ipilimumab and tremelimumab, enhance T-cell priming and expansion through mechanisms complementary to PD-1 or PD-L1 blockade. Their combination with nivolumab or durvalumab, respectively, has produced durable tumor responses and improved overall survival in patients with unresectable or advanced HCC [84,85,87].

Although immune checkpoint inhibitors have substantially improved the treatment landscape for advanced HCC, durable clinical responses remain limited by primary and acquired resistance, immune-related toxicities, and the profound molecular and immunologic heterogeneity of the disease. These challenges underscore the need to identify novel therapeutic targets and rational combination strategies capable of overcoming immune evasion and improving long-term patient outcomes.

## 5. Complexity and Challenges in Treating HCC

Despite substantial advances in locoregional and systemic therapies, HCC remains one of the most challenging malignancies to treat because of its remarkable biological and clinical complexity. Most HCC cases arise in the setting of chronic liver disease, often accompanied by cirrhosis [3]. Cirrhosis, characterized by extensive fibrosis and distortion of the hepatic architecture, not only increases the risk of hepatocarcinogenesis but also compromises hepatic functional reserve, thereby limiting patients’ tolerance to surgical resection, locoregional therapies, and systemic treatments. Consequently, the coexistence of liver dysfunction and malignancy substantially restricts therapeutic options and increases the risk of treatment-related complications [8]. Beyond the challenges imposed by underlying liver disease, HCC is a highly heterogeneous malignancy driven by the complex interplay of genetic, epigenetic, metabolic, and environmental factors that shape tumor initiation, progression, and therapeutic response [6]. This extensive molecular and cellular heterogeneity complicates the identification of universal therapeutic targets and contributes to treatment resistance, tumor recurrence, and disease progression. The major biological complexities and clinical challenges that limit the efficacy of current HCC therapies are discussed in the following sections.

### 5.1. Genetic and Epigenetic Alterations in HCC

HCC arises from diverse etiologies that shape the molecular landscape of individual tumors and influence therapeutic responses. For example, HBV-associated HCC exhibits a genomic profile distinct from that of MASLD-associated HCC, reflecting differences in the underlying mechanisms of hepatocarcinogenesis [3,6]. Importantly, HCC risk factors rarely act independently. Instead, chronic viral hepatitis, excessive alcohol consumption, MASLD, obesity, and metabolic disorders frequently coexist and synergistically accelerate liver injury, fibrosis, and malignant transformation, thereby increasing both the incidence and biological complexity of HCC [5,6,8].

Inherited genetic susceptibility further contributes to HCC development. Several genetic variants involved in lipid metabolism influence disease susceptibility across both MASLD and ALD. Among these, the patatin-like phospholipase domain-containing protein 3 (*PNPLA3*) rs738409 C>G variant, which encodes the I148M substitution, is the strongest common genetic risk factor for progressive steatotic liver disease and is associated with increased hepatic steatosis, inflammation, fibrosis, and HCC development [142–146]. Variants in transmembrane 6 superfamily member 2 (*TM6SF2*) and membrane-bound O-acyltransferase domain-containing 7 (*MBOAT7*) further increase hepatic lipid accumulation and disease progression [147–150]. In contrast, the loss-of-function hydroxysteroid 17-beta dehydrogenase 13 (*HSD17B13*) rs72613567 T>A variant is associated with reduced susceptibility to ALD and HCC [151]. Collectively, these findings highlight the shared genetic susceptibility between MASLD and ALD involving *PNPLA3*, *TM6SF2*, *MBOAT7*, and *HSD17B13*. Variants in these lipid-metabolism genes modify the risk of progressive liver injury, cirrhosis, and HCC, although their effects differ in direction and magnitude, with PNPLA3, TM6SF2, and MBOAT7 generally conferring increased risk and loss-of-function HSD17B13 variants providing partial protection [152–154].

Comprehensive genomic analyses have revealed recurrent somatic alterations that drive HCC initiation and progression (Figure 1). Among the most frequently mutated genes is tumor protein p53 (*TP53*), a critical tumor suppressor whose loss- and gain-of-function mutations promote genomic instability, tumor initiation, and disease progression [3]. Importantly, distinct TP53 mutations can undergo convergent evolution, whereby independent tumor clones acquire different mutations that produce similar oncogenic phenotypes [3]. Mutations in catenin beta 1 (*CTNNB1*), which encodes the central effector of the Wnt/β-catenin signaling pathway, also represent common early driver events in HCC and similarly exhibit convergent evolutionary patterns [3]. In addition, activating mutations in the telomerase reverse transcriptase (*TERT*) promoter occur early during hepatocarcinogenesis, promoting telomerase reactivation, cellular immortalization, and tumor progression [3]. TERT activation may arise through promoter mutations or HBV integration into the TERT locus, highlighting the close interplay between genetic and epigenetic mechanisms in HCC development [3]. Alterations in chromatin-remodeling genes, including AT-rich interaction domain 1A (*ARID1A*) and AT-rich interaction domain 1 B (*ARID1B*), further contribute to hepatocarcinogenesis by altering chromatin accessibility and transcriptional programs [3]. Moreover, increased expression of the multidrug resistance protein 1 (*MDR1*) gene has been reported in some HBV-associated HCCs, potentially contributing to enhanced drug efflux and therapeutic resistance [155].

Beyond somatic mutations, epigenetic dysregulation represents another hallmark of HCC. Aberrant DNA methylation is detectable in cirrhotic liver tissue before tumor formation, supporting the concept of a field cancerization effect, in which widespread epigenetic alterations predispose chronically injured liver tissue to malignant transformation [3,16]. Several genes, including suppressor of cytokine signaling 2 (*SOCS2*) and ubiquitin D (*UBD*), exhibit altered methylation patterns in both preneoplastic lesions and established HCC, suggesting that epigenetic reprogramming contributes to the earliest stages of hepatocarcinogenesis [3]. Together with chromatin remodeling and transcriptional dysregulation, these epigenetic alterations further increase molecular heterogeneity among HCC tumors.

Despite major advances in defining the genomic landscape of HCC, translating these discoveries into effective targeted therapies remains a significant challenge. Frequently altered genes, including *TP53*, *CTNNB1*, AXIS inhibition protein 1 (*AXIN1*), TSC complex subunit 2 (*TSC2*), *ARID1A*, and *ARID1B*, remain difficult to target pharmacologically using current therapeutic approaches [28,117,156]. Moreover, HCC is characterized by extensive genetic and epigenetic alterations, with distinct molecular alterations that arise depending on the underlying etiology [3,5,117]. Such heterogeneity contributes significantly to variability in the therapeutic response, as treatments targeting specific pathways may be ineffective in tumors lacking these molecular alterations. For example, TP53-mutant HCCs, which are commonly associated with HBV-related and aflatoxin-exposed tumors, and CTNNB1-mutant HCCs, which are frequently observed in nonviral and some MASLD-associated molecular subclasses, display distinct signaling, immune, and metabolic features that may influence their sensitivity to systemic therapies [157–159]. Collectively, these findings highlight the intricate interplay among genetic, epigenetic, metabolic, and environmental factors that drive hepatocarcinogenesis and underscore the need for precision medicine strategies that account for the molecular diversity of HCC.

### 5.2. Inter- and Intrahepatic Tumor Heterogeneity

HCC exhibits remarkable inter- and intrahepatic tumor heterogeneity, encompassing extensive genetic, epigenetic, phenotypic, and microenvironmental diversity both among different patients and within individual tumors or distinct hepatic lesions [3,6,160]. This heterogeneity represents one of the greatest challenges in HCC management as genetically and phenotypically distinct tumor subclones can respond differently to the same therapy, thereby promoting treatment resistance, tumor progression, and recurrence. Etiologic diversity, genomic instability, epigenetic reprogramming, cancer stem cell plasticity, and clonal evolution under therapeutic pressure collectively contribute to the heterogeneous landscape of HCC.

Intratumoral heterogeneity is evident even within a single lesion, where spatially distinct tumor regions frequently harbor different somatic mutations and signaling pathway alterations [3,6]. For example, subclonal mutations in phosphatidylinositol-4,5-bisphosphate 3-kinase catalytic subunit alpha (*PIK3CA*), which occur in approximately 28–35.6% of HCCs, may influence responsiveness to inhibitors targeting the PI3K-AKT-mTOR signaling pathway [6]. As a result, therapies directed against a single molecular target may eliminate sensitive tumor populations while allowing resistant subclones to survive and expand, ultimately leading to disease progression and therapeutic failure.

Beyond genetic diversity, HCC also displays profound epigenetic heterogeneity [3]. Variability in DNA methylation patterns, particularly at cytosine-phosphate-guanine (CpG) sites, has been reported within individual tumors, with methylation heterogeneity reaching as high as 64% [3,6]. Aberrant hypermethylation of genes regulating cell proliferation, differentiation, apoptosis, and other essential cellular processes further increase functional diversity among tumor cells and contributes to differential responses to systemic therapies.

The extensive spatial and molecular heterogeneity of HCC presents major obstacles to precision oncology. Conventional single-region biopsies, which remain the standard approach for histopathological diagnosis and molecular profiling, often fail to capture the full spectrum of genomic and epigenomic alterations present within a tumor or across multiple hepatic lesions. Consequently, clinically relevant subclones may be overlooked, leading to incomplete molecular characterization, inaccurate assessment of tumor aggressiveness, and suboptimal therapeutic selection. For instance, a single-region biopsy might miss a subclone harboring a *PIK3CA* mutation, potentially leading to inappropriate therapeutic decisions such as ineffective use or non-use of mTOR-targeted inhibitors [6]. These limitations have stimulated growing interest in multiregional tissue sampling, liquid biopsy approaches, and single-cell and spatial multi-omics technologies, which offer a more comprehensive view of tumor evolution and intratumoral heterogeneity and may ultimately improve precision medicine strategies for HCC.

### 5.3. Cancer Stems Cells Drive Therapeutic Resistance and Tumor Recurrence in HCC

Cancer stem cells (CSCs) represent a small but highly tumorigenic subpopulation of HCC cells that contributes substantially to disease progression, therapeutic resistance, and tumor recurrence (Figure 1). Although CSCs comprise only a minor fraction of the tumor, they possess stem-like properties, including self-renewal, multilineage differentiation, and tumor-initiating capacity, enabling them to sustain tumor growth and regenerate heterogeneous tumor cell populations following treatment [161,162]. Multiple CSC markers have been identified in HCC and are associated with tumor aggressiveness and unfavorable clinical outcomes. Cell populations expressing CD45^−^CD90^+^, particularly the highly metastatic CD90^+^CD44^+^ subset, correlate with increased tumor growth, vascular invasion, and metastatic dissemination [163]. Elevated expression of cytokeratin 19 (CK19), ATP-binding cassette subfamily G member 2 (ABCG2), prominin-1 (CD133/PROM1), and NESTIN has been associated with enhanced angiogenesis, stemness, and poor prognosis [164].

At the molecular level, CSC maintenance is regulated by stemness-associated transcription factors, including octamer-binding transcription factor 4 (OCT4) and polycomb group ring finger protein BMI1 (BMI1), both of which are highly expressed in HCC tissues and circulating CSCs and promote self-renewal and hepatocarcinogenesis [163,164]. CSCs exhibit intrinsic resistance to chemotherapy, molecularly targeted therapies, and radiotherapy through multiple mechanisms, including cellular quiescence, enhanced DNA damage repair, increased drug efflux, metabolic adaptation, resistance to apoptosis, and activation of pro-survival signaling pathways. For example, CSCs expressing high levels of Frizzled-10 (FZD10) activate the Wnt/β-catenin–c-Jun–MEK/ERK signaling cascade, promoting stem cell expansion and resistance to lenvatinib [165]. In addition, CSCs actively remodel the tumor microenvironment by interacting with immune cells, stromal cells, and extracellular matrix components to establish an immunosuppressive niche that facilitates immune evasion, angiogenesis, and sustained tumor growth.

The remarkable plasticity and persistence of CSCs allow them to survive conventional therapies and repopulate tumors after treatment, making them a major obstacle to durable clinical responses. Consequently, therapeutic strategies that target CSC-specific signaling pathways, disrupt CSC–tumor microenvironment interactions, or eliminate CSC populations in combination with conventional therapies may represent a promising approach to overcoming therapeutic resistance and reducing tumor recurrence in HCC.

### 5.4. Interpatient Variability Contributes to Therapeutic Resistance in HCC

Clinical responses to currently available HCC therapies vary substantially among patients, reflecting the marked biological heterogeneity of the disease and the complexity of drug resistance. HCC exhibits both primary (intrinsic) and acquired resistance to systemic therapies, with many patients demonstrating limited initial responses or developing resistance after an initial period of clinical benefit [5,9,117]. These observations underscore the need for predictive biomarkers and personalized therapeutic strategies. Therapeutic resistance arises through multiple, often overlapping, mechanisms (Figure 1). These include impaired drug uptake, increased drug efflux, altered drug metabolism, activation of compensatory signaling pathways, enhanced DNA damage repair, dysregulated apoptosis, metabolic reprogramming, and adaptive interactions between tumor cells and the surrounding microenvironment [166–170]. Under therapeutic pressure, HCC cells continue to evolve by acquiring additional genetic and epigenetic alterations that promote clonal selection and drug resistance.

Resistance to antiangiogenic therapies exemplifies the adaptive nature of HCC. Sorafenib resistance is frequently mediated by activation of alternative proangiogenic pathways, particularly FGF/FGFR signaling [5,171]. Prolonged sorafenib exposure can also induce epithelial-to-mesenchymal transition (EMT) through activation of the PI3K-AKT-snail family transcriptional repressor 1 (SNAIL) pathway, thereby promoting invasion, metastasis, and therapeutic resistance [6,171]. Likewise, acquired lenvatinib resistance in HCC has been linked to activation of the EGFR-STAT3 signaling axis, which enhances expression of the multidrug efflux transporter ATP-binding cassette subfamily B member 1 (ABCB1, also known as P-glycoprotein), thereby reducing intracellular lenvatinib accumulation and diminishing its antitumor activity [126,172].

Intrinsic molecular alterations also influence treatment responses. Activation of tumor-intrinsic Wnt/β-catenin signaling, most through gain-of-function *CTNNB1* mutations, has been strongly associated with an immune-excluded tumor microenvironment and resistance to immune checkpoint blockade in HCC [173,174]. In contrast, Wnt/β-catenin alterations have not consistently predicted clinical responses to sorafenib. These findings suggest that Wnt/β-catenin activation may have greater potential as a biomarker of immunotherapy resistance than as a predictor of multikinase inhibitor response, although prospective validation is still required.

The tumor microenvironment further contributes to therapeutic failure through reciprocal interactions among tumor cells, immune cells, stromal cells, endothelial cells, and extracellular matrix components that promote tumor survival and suppress antitumor immunity [6,79]. In addition, treatment-induced autophagy provides metabolic substrates that enable tumor cells to survive therapeutic stress, further reducing treatment efficacy [175–177]. Collectively, these adaptive mechanisms contribute substantially to the wide interpatient variability observed in HCC treatment responses.

### 5.5. Limitations of Targeted Therapies and Predictive Biomarkers for Therapeutic Stratification in HCC

Despite significant advances in systemic therapy, targeted treatment for HCC remains constrained by modest efficacy, limited durability of response, and restricted applicability, particularly in patients with advanced disease. Although multikinase inhibitors and immune checkpoint inhibitors have improved overall survival, their clinical benefits remain incremental and are frequently accompanied by treatment-related toxicities [5,8,9]. A major limitation is the relatively low prevalence of actionable genomic alterations in HCC. Current genomic profiling studies indicate that only approximately 25% of patients harbor potentially targetable alterations [5]. These include *TSC1/2* inactivating mutations (8.5%), *FGF19* amplifications (6.3%), *MET* amplification (1.5%), and *IDH1* mutations (<1%) [116]. However, these alterations occur infrequently and, in many cases, lack clinically validated targeted therapies with demonstrated survival benefit.

Another major challenge is the absence of robust predictive biomarkers to guide therapeutic selection. Without reliable biomarkers, clinicians cannot accurately identify patients most likely to benefit from specific targeted or immunotherapeutic agents, resulting in suboptimal treatment selection and highly variable clinical outcomes [116]. The identification of predictive biomarkers therefore remains essential for advancing precision medicine in HCC.

Beyond identifying novel molecular targets, improving tumor-selective drug delivery represents another promising strategy to enhance therapeutic efficacy while minimizing systemic toxicity. Recently, a soluble triantennary N-acetylgalactosamine (GalNAc)-camptothecin prodrug targeting the asialoglycoprotein receptor (ASGPR) demonstrated enhanced liver-specific drug delivery, improved tumor accumulation, reduced systemic toxicity, and potent antitumor activity in preclinical HCC models [178]. Moreover, activation of the cyclic GMP-AMP synthase-stimulator of interferon genes (cGAS-STING) pathway and increased CD8^+^ T-cell infiltration suggest that liver-targeted drug delivery systems may also augment antitumor immunity, providing a promising complementary strategy for HCC therapy [178].

Collectively, the therapeutic landscape of HCC remains constrained by late-stage diagnosis, underlying liver dysfunction, extensive molecular heterogeneity, rapid emergence of therapeutic resistance, and the lack of robust predictive biomarkers. Future advances will require integrated precision medicine approaches that combine comprehensive genomic and multi-omics profiling, improved characterization of the tumor microenvironment, biomarker-guided therapeutic stratification, innovative liver-targeted drug delivery platforms, and the development of therapies that target both oncogenic signaling pathways and post-transcriptional regulatory networks. Such multidisciplinary strategies are expected to improve therapeutic efficacy and expand personalized treatment options for patients with HCC.

## 6. Targeting RNA-Binding Protein HuR in HCC: Mechanisms and Therapeutic Potential

An emerging class of targets includes RNA-binding proteins, particularly human antigen R (HuR) protein, encoded by the ELAV like RNA binding protein 1 (*ELAVL1*) gene [179]. HuR is a ubiquitously expressed RNA-binding protein that belongs to the RNA recognition motif (RRM) superfamily and functions as a master post-transcriptional regulator of gene expression [179]. HuR preferentially binds to AU-rich elements (AREs) located within the 3′ untranslated regions (3′UTRs) of target mRNAs, thereby regulating their stability, translation, and subcellular localization [179]. Through these actions, HuR controls numerous biological processes, including cell cycle progression, cellular differentiation, cytokine production, signal transduction, stress responses, inflammation, metabolism, and apoptosis [180,181].

HuR is expressed in virtually all tissues, although its abundance and subcellular localization vary depending on tissue type, developmental stage, and physiological or pathological conditions [182]. Consistent with its essential role in maintaining cellular homeostasis, complete loss of *HuR* is embryonically lethal. Global deletion of *Elavl1* results in severe developmental abnormalities, including defective extraembryonic placental formation, impaired skeletal ossification, limb fusion, and asplenia, ultimately leading to embryonic nonviability [183]. These findings underscore the indispensable role of HuR in normal development and tissue homeostasis.

Increasing evidence indicates that dysregulated HuR expression and activity contribute to multiple hallmarks of cancer, including sustained proliferation, metabolic reprogramming, resistance to apoptosis, inflammation, invasion, metastasis, and therapeutic resistance. In the liver, HuR plays a critical role in maintaining hepatic metabolic homeostasis under physiological conditions, whereas its aberrant activation promotes hepatocarcinogenesis and HCC progression [179,181]. In this section, we first summarize the physiological functions of HuR in maintaining liver homeostasis, followed by its dysregulation during HCC development and progression, and conclude by discussing emerging therapeutic strategies targeting HuR for the treatment of HCC.

### 6.1. HuR Regulation of Hepatic Homeostasis in Normal Liver

In hepatocytes, HuR stabilizes mRNAs of key metabolic proteins, thereby influencing lipid metabolism and the function of detoxification enzymes [184–186]. It also controls the translation of mRNAs for growth factors and cytokines, which affect cell proliferation and responses to external stimuli [187,188]. Disruption of HuR function can destabilize target mRNAs, resulting in their degradation and the subsequent impairment of normal liver function [189]. Moreover, dysregulation of HuR may impair the ability of cells to respond to stress, making liver cells more vulnerable to damage and less resilient to environmental challenges. This vulnerability can lead to uninhibited proliferation and survival of mutated cells within the hepatocytes. In addition to hepatocytes, HuR also plays an important role in non-parenchymal cells. Increased HuR activity in hepatic stellate cells (HSCs) contributes to excessive extracellular matrix production, leading to liver fibrosis [190]. Importantly, both increased and decreased HuR activity can disrupt hepatic homeostasis, indicative of its function as a finely tuned regulator of mRNA stability and cellular equilibrium. Collectively, these observations underscore the critical role of HuR in maintaining liver homeostasis and highlight the consequences of its dysregulation in liver pathophysiology.

### 6.2. Dysregulation of HuR Drives Cancer Development

Clinically, HuR has been implicated in malignant phenotypes and patient prognosis in various cancers, including oral, colorectal, gallbladder, renal, pancreatic, lung, breast, and HCC [191]. These observations suggest that HuR is a promising therapeutic target for several malignancies, including glioblastoma, breast cancer, and HCC [192,193]. HuR overexpression has also been associated with drug resistance in some cancers. For example, in glioma models, silencing HuR reduces tumor volume, whereas overexpression of HuR promotes chemoresistance by regulating anti-apoptotic pathways, including B-cell lymphoma 2 (Bcl-2) [193]. HuR promotes cancer cell survival by regulating the expression of anti-apoptotic proteins such as Bcl-2 and X-linked inhibitor of apoptosis protein (XIAP), triggering autophagy and autophagosome formation [194]. In HepG2 hepatoblastoma cells, HuR has been shown to reduce radiosensitivity by stabilizing mitochondrial transcription factor A (*TFAM*) mRNA and maintaining mitochondrial function under stress [195].

Both the expression level and subcellular location of HuR are critical determinants of oncogenic function. Aberrant HuR activity has been linked to almost every hallmark of cancer: sustaining proliferative signaling, evading suppression of growth, promoting invasion and metastasis, enabling replicative immortality, inducing angiogenesis, resisting cell death, deregulating cellular energetics, promoting tumor-associated inflammation, and avoiding immune destruction [196–204]. Upregulation and increased cytoplasmic localization of HuR have been associated with advanced tumor stage and poor survival in HCC, while mechanistic studies indicate that cytoplasmic HuR promotes tumor-cell invasion and metastatic progression by stabilizing pro-tumorigenic transcripts [180,181,205,206]. Nucleocytoplasmic shuttling of HuR is regulated by cellular stress and is mediated through nuclear pore transport, enabling HuR to stabilize target mRNAs in the cytoplasm [207,208]. Thus, dysregulation of HuR localization and activity contributes to divergent oncogenic outcomes and complicates its therapeutic targeting.

HuR function is tightly regulated by multiple signaling pathways that control post-translational modifications and subcellular trafficking. These include mitogen-activated protein kinases (MAPKs), AMP-activated kinase (AMPK), cyclin-dependent kinase 1 (CDK1), cell cycle checkpoint kinase 2 (CHK2), coactivator-associated arginine methyltransferase 1 (CARM1), and protein kinase C (PKC) [209]. For instance, P38 MAPK phosphorylates HuR protein at Thr-118 and promotes its cytoplasmic translocation to stabilize target mRNAs such as *p21* and prostaglandin-endoperoxide synthase 2 (*PTGS2*, also known as *COX2*) during inflammation [210]. CDK1 phosphorylates HuR at S202 and facilitates HuR interaction with 14-3-3 for nuclear retention [211,212], whereas CDK1 inhibition promotes HuR translocation to the cytoplasm and stabilizes cyclin-dependent kinase inhibitor 1A (*CDKN1A*) mRNA, a tumor suppressor gene [213]. During DNA damage, CHK2 is activated, leading to the phosphorylation of S88, S100, and Thr 118 residues of HuR, modulating the RNA-binding activity of HuR [214]. In parallel, CARM1 methylates HuR at the arginine residue R217 to promote mRNA stability of HuR targets [215]. In addition, PKC phosphorylates HuR to promote its cytoplasmic exportation and control mRNA stability to encode proteins related to various cancer pathways [216,217]. Specifically, PKC phosphorylates the S158 and S221 residues of HuR, leading to enhanced binding and stabilization of *COX2* mRNA and prostaglandin biosynthesis, thereby enhancing downstream inflammatory and tumor-promoting signaling [218].

Collectively, these regulatory pathways orchestrate HuR localization and function, and their dysregulation contributes to the progression of chronic liver disease and hepatocarcinogenesis. The complexity of HuR regulation—spanning expression, localization, and post-translational modifications—underscores its central role in tumor biology and highlights both the promise and challenges of targeting HuR as a therapeutic strategy.

### 6.3. Therapeutic Targeting of HuR in HCC

Advanced HCC remains difficult to treat owing to intrinsic heterogeneity and the frequent emergence of drug resistance, both of which limit the durability of current therapies [8,9]. Given the central role of post-transcriptional regulation in these processes, targeting the RNA-binding protein, HuR, has emerged as a promising therapeutic strategy. HuR overexpression has been identified in various tumor types, including HCC, where it contributes to tumor progression, poor prognosis, and resistance to therapy by stabilizing the mRNAs encoding oncogenic and pro-survival factors [181,191,219].

HuR regulates a broad network of transcripts that collectively drive hepatocarcinogenesis (Figure 1). Some HuR targets include—but are not limited to—methionine adenosyltransferase 2A (*MAT2A*), murine double minute 2 (*MDM2*), Fas cell surface death receptor (*FAS*), *BCL-2*, and *XIAP* [181]. For example, HuR binds to the 3′-UTR of *MAT2A* and stabilizes its transcript, contributing to increased MAT2A expression during hepatocyte dedifferentiation and hepatocarcinogenesis [220]. With *MDM2*—a cell cycle regulator—HuR directly binds and stabilizes *MDM2* mRNA, whereas MDM2 reciprocally stabilizes HuR through NEDDylation, forming a positive regulatory circuit that enhances the expression of cell cycle regulators and promotes tumor cell proliferation [221,222]. In contrast, HuR binds to the 3′-UTR of *FAS* mRNA and represses its translation, thereby reducing FAS protein and protecting HCC cells from FAS-mediated apoptosis [180]. Conversely, silencing *HuR* restores FAS expression and sensitizes HCC cells to Fas ligand (FASL)-induced apoptosis [180].

As previously stated, the oncogenic activity of HuR is tightly linked to its subcellular localization and post-translational regulation. For instance, phosphorylation of liver kinase B1 (LKB1) at Ser428 promotes HuR cytoplasmic localization in hepatoma cells, leading to stabilization of herpesvirus-associated ubiquitin-specific protease (*HAUSP*) mRNA, which modulates the HAUSP-p53-MDM2 regulatory axis and reduces susceptibility to stress-induced apoptosis [223,224]. *HuR* silencing in a cholestatic liver injury model reduced the expression of chemoattractant and pro-inflammatory genes, leading to decreased oxidative stress, macrophage infiltration, liver damage, and fibrosis [190]. In hepatic stellate cells, HuR stabilizes beclin 1 (*BECN1*) mRNA and promotes autophagy-dependent ferritin degradation, thereby increasing intracellular iron availability and ferroptosis. This HuR–BECN1–ferritinophagy axis highlights the context-dependent interplay between autophagy and ferroptosis and may provide opportunities for therapeutic intervention [225,226]. In addition, HuR participates in regulatory networks involving long non-coding RNAs such as MIR22HG. In HCC cells, HuR binds and stabilizes *MIR22HG*, whereas MIR22HG reciprocally alters HuR subcellular localization and competitively limits its association with oncogenic target mRNAs, thereby suppressing tumor-cell proliferation, invasion, and metastasis [227]. Moreover, HuR contributes to the activation of oncogenic signaling pathways by stabilizing transcripts encoding key pathway effectors. In HCC, HuR cooperates with the lncRNA UFC1 to stabilize *CTNNB1* mRNA and enhance Wnt/β-catenin signaling [228]. HuR has also been shown to bind the *YAP1* 3′-UTR and maintain expression of the Hippo-pathway effector YAP1, although this mechanism has been characterized primarily in pancreatic cancer [229]. Collectively, these diverse functions position HuR as a central regulator of proliferation, survival, angiogenesis, immune recognition, invasion, and metastasis [230]. Consistently, knockdown of HuR in cancer cells resulted in decreased tumor cell growth and impaired invasion capabilities, establishing HuR as a promising target for inhibiting cancer invasion, metastasis, and tumor progression [194].

Efforts to inhibit HuR therapeutically have led to the development of small-molecule inhibitors and RNA-based strategies. High-throughput screening identified KH-3 as a novel small molecule that disrupts HuR-mRNA interactions [231]. KH-3 competitively binds to HuR, inhibiting its function and leading to the decay of HuR target mRNA and reduced translation (Figure 1). One direct target affected by KH-3 in breast cancer is forkhead box Q1 (*FOXQ1*), a transcription factor associated with proliferation, EMT, and cancer stemness [232–235]. FOXQ1 also regulates genes involved in cancer invasion and metastasis, such as *SNAIL* and laminin subunit alpha 4 (*LAMA4*) [236,237].

Beyond KH-3, small molecular compounds, such as MS-444, N-benzylcantharidinamide, latrunculin A, and blebbistatin, have shown inhibitory effects on HuR cytoplasmic translocation, reducing its ability to stabilize target mRNAs [181,219]. The small-molecule compound CMLD2, similar to KH-3, directly inhibits HuR binding to target mRNAs [181,195,219]. RNA-based approaches may disrupt HuR function by using decoy RNAs that competitively sequester HuR and prevent its interaction with selected target transcripts [197,238]. In addition, tumor-targeted folate- or transferrin-conjugated nanocarriers have also enabled selective delivery of HuR siRNA, resulting in HuR depletion and suppression of lung and ovarian tumor growth in preclinical models [239–241].

Despite these advances, significant challenges remain in translating HuR-targeted therapies into clinical practice (Table 3). These include achieving selective inhibition without disrupting the physiological functions of HuR in normal tissues, overcoming compensatory signaling pathways, and identifying patient populations most likely to benefit from HuR-targeted interventions. In addition, current knowledge regarding the therapeutic potential of HuR in HCC is still limited, as many mechanistic insights into HuR function have been derived from studies of normal liver physiology or other cancer types rather than HCC-specific investigations. Nevertheless, the accumulating evidence supporting the critical role of HuR in maintaining liver homeostasis and regulating multiple oncogenic pathways provides a strong biological rationale for targeting HuR in HCC. Further HCC-specific mechanistic and preclinical studies are needed to better define the role of HuR in hepatocarcinogenesis and to facilitate the development of clinically effective and safe HuR-targeted therapies.

## 7. Beyond HuR: Additional Emerging Therapeutic Targets in HCC

While targeting HuR represents a promising strategy, the multifactorial nature of HCC necessitates the exploration of additional therapeutic targets to overcome tumor heterogeneity and resistance. Beyond HuR, several well-established oncogenic pathways continue to serve as important HCC targets. FGFR4 signaling is frequently upregulated in HCC and promotes tumor growth, angiogenesis, and metastasis [3,124]. Targeting the FGF19–FGFR4 signaling axis has emerged as a promising biomarker-driven therapeutic strategy for HCC. Selective FGFR4 inhibitors, including fisogatinib (BLU-554), have demonstrated encouraging clinical activity in patients with FGF19-positive tumors, and newer FGFR4-targeted agents and combination therapies continue to be evaluated in clinical studies [242–244]. *TERT* promoter mutations are among the most frequent genetic alterations in HCC and represent an early event in hepatocarcinogenesis [157,245]. Consequently, direct inhibition of TERT or disruption of pathways regulating telomerase activity has emerged as a promising therapeutic strategy. In particular, anti-TERT antisense oligonucleotides induced telomere shortening, DNA damage, proliferative arrest, and apoptosis in HCC cell lines and suppressed tumor growth in a xenograft model, providing direct preclinical evidence that telomerase addiction is therapeutically actionable in HCC [246]. Additional experimental approaches targeting TERT expression or TERT-expressing tumor cells have also demonstrated antitumor activity, although further validation and safety studies are required before clinical translation [247].

The Wnt/β-catenin pathway is a critical regulator of cell proliferation, differentiation, and tissue homeostasis [248]. Aberrant activation of this pathway via mutations in *CTNNB1* has been associated with tumor proliferation, immune evasion, and resistance to immunotherapy [249]. Thus, targeting the Wnt/β-catenin pathway with small-molecule inhibitors or antibodies is an active area of research in HCC [5,133]. Parallel signaling pathways, including RAS/MAPK and PIK3/AKT, are frequently dysregulated in HCC and contribute to tumor progression and drug resistance [118]. Targeting these pathways, either alone or in combination with existing therapies, may improve the treatment efficacy.

Epigenetic regulation is another promising area of therapeutic intervention. DNA methylation, histone modification, and chromatin remodeling play key roles in the initiation and progression of HCC [3,8]. Agents targeting DNA methyltransferases and histone deacetylases are currently under investigation for their ability to reverse abnormal epigenetic states and inhibit tumor growth [3,6].

The tumor microenvironment has emerged as a critical determinant of HCC progression and therapeutic response. Interactions between tumor cells, immune cells, hepatic stellate cells, and endothelial cells create a dynamic environment that supports tumor growth, fibrosis, and immune evasion. Targeting components of this microenvironment, including angiogenesis, immune checkpoints, and fibrotic signaling pathways, has shown clinical benefits and remains an active area of research [6]. Collectively, these alternative targets reflect the multifaceted nature of HCC and underscore the need for integrated therapeutic strategies. Approaches that simultaneously address tumor-intrinsic signaling, post-transcriptional regulation, and the surrounding microenvironment may offer the greatest potential for improving patient outcomes.

## 8. Conclusions and Future Perspectives

HCC remains one of the most difficult malignancies to treat because of its profound biological complexity, frequent presentation at advanced stages, and limited responsiveness to existing therapies. A defining feature of HCC is its extensive inter- and intratumoral heterogeneity, encompassing genetic, epigenetic, metabolic, phenotypic, and microenvironmental diversity. This heterogeneity is shaped by distinct etiologies, including chronic viral hepatitis, ALD, MASLD, inherited metabolic disorders, and genetic susceptibility. As a result, tumors arising in different patients—and even separate lesions or regions within the same patient—may exhibit markedly different molecular profiles and therapeutic vulnerabilities. This diversity complicates diagnosis, prognostic assessment, biomarker development, and treatment selection, while limiting the effectiveness of uniform or single-target therapeutic strategies.

Therapeutic resistance further compounds these challenges. HCC cells may display intrinsic resistance because of pre-existing genetic, epigenetic, or metabolic features, while acquired resistance can emerge through clonal evolution, activation of compensatory signaling pathways, enhanced drug efflux, altered drug metabolism, improved DNA damage repair, and suppression of apoptosis. The tumor microenvironment also plays a central role by promoting angiogenesis, immune evasion, stromal support, and metabolic adaptation, thereby protecting tumor cells from treatment-induced injury. In addition, interpatient differences in drug absorption, metabolism, distribution, and clearance can contribute to pharmacokinetic variability and inconsistent clinical responses.

These challenges underscore the need to move beyond uniform treatment paradigms toward integrated, biomarker-driven, and personalized therapeutic strategies. Curative approaches, including surgical resection, local ablation, and liver transplantation, remain restricted largely to patients with early-stage disease. Multikinase inhibitors such as sorafenib and lenvatinib, as well as immune checkpoint inhibitor-based combinations such as atezolizumab plus bevacizumab, have expanded treatment options for advanced HCC; however, their benefits remain limited by toxicity, primary resistance, and the eventual emergence of acquired resistance. Future therapeutic development should therefore focus on rational combinations that simultaneously target tumor-intrinsic pathways, cancer stem cells, immune suppression, angiogenesis, metabolic vulnerabilities, and the surrounding tumor microenvironment.

Technological advances are also expected to accelerate the transition toward precision medicine. Single-cell sequencing, spatial transcriptomics, multi-omics profiling, and liquid biopsy approaches can provide a more comprehensive view of tumor heterogeneity, clonal evolution, and treatment response. These platforms may facilitate the identification of predictive biomarkers, improve patient stratification, and enable longitudinal monitoring of emerging resistance. In parallel, epigenetic therapies, RNA-based approaches, and liver-targeted drug delivery systems offer additional opportunities to improve therapeutic specificity while reducing systemic toxicity.

Among emerging molecular targets, HuR is particularly promising because it functions as a central post-transcriptional regulator of mRNA stability and translation. Through coordinated control of transcripts involved in proliferation, survival, inflammation, angiogenesis, metabolism, and therapeutic resistance, HuR may integrate multiple oncogenic pathways that are otherwise difficult to target simultaneously. Pharmacological HuR inhibitors, including KH-3, have shown the potential to disrupt these regulatory networks and enhance tumor sensitivity to treatment. Nevertheless, additional HCC-specific mechanistic studies, rigorous preclinical validation, and careful assessment of therapeutic safety and tissue selectivity will be required before HuR-targeted therapies can be translated into clinical practice.

In summary, meaningful improvement in HCC outcomes will require a comprehensive strategy that integrates molecular profiling, predictive biomarkers, rational combination therapy, tumor-selective drug delivery, and longitudinal monitoring of treatment response. Continued translation of mechanistic discoveries into clinically actionable interventions will be essential to overcome tumor heterogeneity, therapeutic resistance, and the limitations of current treatment approaches. Targeting broad regulatory nodes such as HuR, particularly in combination with established and emerging therapies, may provide a promising path toward more durable and personalized treatment of HCC.

## Figures and Tables

**Table 1.** Barcelona Clinic Liver Cancer (BCLC) staging system for hepatocellular carcinoma.

BCLC Stage	Tumor Characteristics	Liver Function/Performance Status	Recommended Treatment	Median Survival (Approx.)
0 (Very Early)	Single nodule ≤ 2 cm; no vascular invasion or metastasis	Child–Pugh A; ECOG 0	Surgical resection, radiofrequency ablation (RFA), or microwave ablation (MWA)	>5 years
A (Early)	Single tumor or up to 3 nodules ≤ 3 cm; no vascular invasion or metastasis	Child–Pugh A–B; ECOG 0	Surgical resection, liver transplantation, or local ablation	>5 years
B (Intermediate)	Multinodular disease without vascular invasion or extrahepatic spread	Preserved liver function; ECOG 0	Transarterial chemoembolization (TACE); selected patients may receive TACE plus systemic therapy	~2–3 years
C (Advanced)	Portal vein invasion, lymph node involvement, or extrahepatic metastasis	Child–Pugh A–B; ECOG 1–2	Systemic therapy (immune checkpoint inhibitors, multikinase inhibitors, targeted therapies)	~1–2 years
D (Terminal)	End-stage disease	Child–Pugh C and/or ECOG > 2	Best supportive care and symptom management	<6 months

The BCLC staging system integrates tumor burden, liver function (Child–Pugh score), and patient performance status (ECOG) to stratify HCC into five clinical stages (0–D), each associated with recommended treatment strategies and expected prognosis. Because it combines prognostic assessment with therapeutic guidance, the BCLC system is the most widely used staging algorithm in clinical practice and is recommended by major international liver societies. Abbreviations: BCLC, Barcelona Clinic Liver Cancer; ECOG, Eastern Cooperative Oncology Group; TACE, transarterial chemoembolization; RFA, radiofrequency ablation; MWA, microwave ablation.

**Table 2.** Systemic therapies currently approved by the U.S. Food and Drug Administration for hepatocellular carcinoma.

Therapeutic Agent or Regimen	Drug Class and Principal Targets	FDA-Approved HCC Indication	Route	Initial HCC Approval
Sorafenib (Nexavar)	Oral multikinase inhibitor targeting RAF, VEGFR1–3, PDGFR-β, KIT, FLT3, and RET	Unresectable HCC	Oral	2007
Lenvatinib (Lenvima)	Oral multikinase inhibitor targeting VEGFR1–3, FGFR1–4, PDGFR-α, KIT, and RET	First-line treatment of unresectable HCC	Oral	2018
Atezolizumab plus bevacizumab (Tecentriq plus Avastin)	PD-L1 immune checkpoint inhibitor plus VEGF-A-neutralizing antibody	Unresectable or metastatic HCC in patients who have not received prior systemic therapy	Intravenous	2020
Tremelimumab plus durvalumab (Imjudo plus Imfinzi; STRIDE regimen)	CTLA-4 inhibitor plus PD-L1 inhibitor	Unresectable HCC	Intravenous	2022
Nivolumab plus ipilimumab (Opdivo plus Yervoy)	PD-1 inhibitor plus CTLA-4 inhibitor	First-line treatment of unresectable or metastatic HCC	Intravenous	2025
Regorafenib (Stivarga)	Oral multikinase inhibitor targeting VEGFR1–3, TIE2, RAF, PDGFR, FGFR, KIT, and RET	HCC previously treated with sorafenib	Oral	2017
Cabozantinib (Cabometyx)	Oral multikinase inhibitor targeting MET, VEGFR1–3, AXL, RET, KIT, and TIE2	HCC previously treated with sorafenib	Oral	2019
Ramucirumab (Cyramza)	Monoclonal antibody targeting VEGFR2	HCC with serum α-fetoprotein ≥ 400 ng/mL following prior sorafenib treatment	Intravenous	2019
Pembrolizumab (Keytruda)	PD-1 immune checkpoint inhibitor	HCC secondary to hepatitis B in patients who have received prior systemic therapy other than a PD-1- or PD-L1-containing regimen	Intravenous or approved subcutaneous formulation	2024 *

* Pembrolizumab initially received accelerated approval in 2018 for HCC previously treated with sorafenib. Its current HCC-specific labeling applies to patients with hepatitis B-associated HCC who have received prior systemic therapy that did not include a PD-1 or PD-L1 inhibitor. Abbreviations: AFP, α-fetoprotein; CTLA-4, cytotoxic T-lymphocyte-associated protein 4; FDA, U.S. Food and Drug Administration; FGFR, fibroblast growth factor receptor; FLT3, FMS-like tyrosine kinase 3; HCC, hepatocellular carcinoma; MET, mesenchymal–epithelial transition factor; PD-1, programmed cell death protein 1; PD-L1, programmed death-ligand 1; PDGFR, platelet-derived growth factor receptor; RAF, rapidly accelerated fibrosarcoma kinase; RET, rearranged during transfection; STRIDE, single tremelimumab regular interval durvalumab; VEGF, vascular endothelial growth factor; VEGFR, vascular endothelial growth factor receptor.

**Table 3.** Potential safety concerns and strategies to improve the therapeutic window of HuR-targeted therapies.

Potential Limitation or Toxicity Risk	Biological Basis	Potential Clinical Consequence	Possible Mitigation Strategy
On-target toxicity in normal tissues	HuR is ubiquitously expressed.	Systemic inhibition may disrupt normal tissue homeostasis and cause organ-specific toxicities.	Tumor- or liver-targeted delivery, intermittent dosing, and partial rather than complete HuR inhibition
Hepatic toxicity	HuR contributes to normal hepatic metabolic and stress-response pathways.	HuR inhibition may aggravate liver dysfunction in patients with cirrhosis or limited hepatic reserve.	Careful dose escalation, liver-function monitoring, and HCC-selective delivery systems
Off-target and compound-specific effects	Current inhibitors, including KH-3, CMLD-2, and MS-444, remain preclinical and may interact with proteins or pathways other than HuR.	Unanticipated toxicity and difficulty distinguishing on-target from off-target effects	Improved compound selectivity, target-engagement assays, pharmacokinetic studies, and structure-guided optimization
Narrow therapeutic window	The HuR functions required for tumor survival may overlap with those needed for normal-cell homeostasis.	Antitumor doses may approach doses that affect normal tissues	Biomarker-guided patient selection, rational combination therapy, and lower-dose synergistic regimens

## Data Availability

No new data were created or analyzed in this study. Data sharing is not applicable to this article.

## Abbreviations

The following abbreviations are used in this manuscript:

HCC: Hepatocellular carcinoma
HBV: Hepatitis B virus
HCV: Hepatitis C virus
MASLD: Metabolic dysfunction-associated steatotic liver disease
NAFLD: Non-alcoholic fatty liver disease
HKLC: Hong Kong Liver Cancer
CLIP: Cancer of the Liver Italian Program
JIS: Japan integrated staging
AFP: Alpha-fetoprotein
TNM: Tumor, node, metastasis
AJCC: American Joint Committee on Cancer
UICC: Union for International Cancer Control
BCLC: Barcelona clinic liver cancer
ECOG: Eastern Cooperative Oncology Group
ALBI: Albumin-bilirubin
MELD: Model for end-stage liver disease
ROS: Reactive oxygen species
GSH: Glutathione
ALD: Alcoholic liver disease
MASH: Metabolic dysfunction-associated steatohepatitis
CLD: Chronic liver disease
STEAP2: Six-transmembrane epithelial antigen of prostate2
MAPK: Mitogen-activated protein kinase
JNK: C-Jun N-terminal kinase
PSC: Primary sclerosing cholangitis
PBC: Primary biliary cholangitis
TACE: Transarterial chemoembolization
DEB: Drug-eluting beads
PD-1: Programmed cell death protein 1
PD-L1: Programmed cell death ligand 1
MKI: Multikinase inhibitors
VEGF: Vascular endothelial growth factor
VEGFR: Vascular endothelial growth factor
PDGFR: Platelet-derived growth factor receptor
RAF: Raf proto-oncogene
KIT: KIT Proto-oncogene, receptor tyrosine kinase
EGFR: Epidermal growth factor receptor
IrAEs: Immune-related adverse events
CTLA-4: Cytotoxin T-lymphocyte associated protein
TM6SF2: Transmembrane 6 superfamily member 2
PNPLA3: patatin-like phospholipase domain-containing 3
MBOAT7: Membrane bound o-acytransferase domain containing 7
HSD17B13: Hydroxysteroid 17-beta dehydrogenase 13
TP53: Tumor protein 53
CTNNB1: Catenin beta 1
TERT: Telomerase reverse transcriptase
ARID1A: AT-rich interaction domain 1A
ARID1B: AT-rich interaction domain 1B
MDR1: Multidrug resistance 1
SOCS2: Suppressor of cytokine signaling 2
UBD: Ubiquitin like modifier D
TSC1/2: TSC complex subunit 1/2
CSC: Cancer stem cells
CK19: Cytokeratin 19
ABCG2: ATP-binding cassette subfamily G member 2
CD133 (PROM1): Prominin-1
OCT4: Octamer-binding transcription factor 4
BMI1: Polycomb ring finger oncogene
FZD10: Frizzeled-10
FGF: Fibroblast growth factor
FGFR: Fibroblast growth factor receptor
EMT: Epithelial-to-mesenchymal transition
PI3K: Phosphoinositide 3-kinase
AKT: Protein kinase B
STAT: Signal transducer and activator of transcription
ABCB1: ATP binding cassette subfamily B member 1
WNT: Wingless-related integration site
AXIN1: AXIS inhibition protein 1
PFS: Progression free survival
OS: Overall survival
MET: MET proto-oncogene, receptor tyrosine kinase
IDH1: Isocitrate dehydrogenase 1
mTOR: Mechanistic target of rapamycin kinase
CpG: Cytosine-guanine dinucleotides
PIK3CA: Phosphatidylinositol-4,5-bisphosphate 3-kinase catalytic subunit alpha
HuR: Human-antigen R
RRM: RNA recognition motif
UTR: Untranslated region
HSC: Hepatic stellate cells
Bcl-2: B-cell lymphoma 2
XIAP: X-linked inhibitor of apoptosis protein
TFAM: Mitochondrial transcription factor A
MAPK/MEK/ERK: Mitogen-activated protein kinase
AMPK: AMP-activated kinase
CDK1: Cyclin-dependent kinase 1
CHK2: Cell cycle checkpoint kinase 2
CARM1: Coactivator-associated arginine methyltransferase 1
PKC: Protein kinase C
COX2: Cyclooxygenase-2
CDKN1A: Cyclin dependent kinase inhibitor 1A
MAT2A: Methionine adenosyltransferase 2A
MDM2: Murine double minute 2
Fas: Fas cell surface death receptor
LKB1: Liver kinase B1
BECN1: Beclin 1
FOXQ1: Forkhead box Q1
LAMA4: Laminin subunit alpha 4
RAS: Rat sarcoma
HAUSP: Herpesvirus-associated ubiquitin-specific protease
ELAVL1: ELAV like RNA binding protein 1
BMI: Body mass index
CYP1A2/3A4: Cytochrome P450 1A2/3A4
JAK: Janus kinase
RET: Rearranged during transfection
